# Cardiovascular magnetic resonance images with susceptibility artifacts: artificial intelligence with spatial-attention for ventricular volumes and mass assessment

**DOI:** 10.1186/s12968-022-00899-5

**Published:** 2022-11-28

**Authors:** Marco Penso, Mario Babbaro, Sara Moccia, Marco Guglielmo, Maria Ludovica Carerj, Carlo Maria Giacari, Mattia Chiesa, Riccardo Maragna, Mark G. Rabbat, Andrea Barison, Nicola Martini, Mauro Pepi, Enrico G. Caiani, Gianluca Pontone

**Affiliations:** 1 Cardiovascular Imaging Department, Centro Cardiologico Monzino IRCCS, Via C. Parea 4, 20138 Milan, Italy; 2 Department of Electronics, Information and Biomedical Engineering, Politecnico di Milano, Milan, Italy; 3 The BioRobotics Institute and Department of Excellence in Robotics and AI, Scuola Superiore Sant’Anna, Pisa, Italy; 4 Department of Biomedical Sciences and Morphological and Functional Imaging, “G. Martino” University Hospital Messina, Messina, Italy; 5 Loyola University of Chicago, Chicago, IL USA; 6 Edward Hines Jr. VA Hospital, Hines, IL USA; 7 Fondazione Toscana Gabriele Monasterio, Pisa, Italy; 8 Istituto di Elettronica e di Ingegneria dell’Informazione e delle Telecomunicazioni, Consiglio Nazionale delle Ricerche, Milan, Italy

**Keywords:** Deep learning, Cardiac segmentation, Cardiac magnetic resonance, Pacemaker, Cardioverter-defibrillators, Susceptibility artifacts

## Abstract

**Background:**

Segmentation of cardiovascular magnetic resonance (CMR) images is an essential step for evaluating dimensional and functional ventricular parameters as ejection fraction (EF) but may be limited by artifacts, which represent the major challenge to automatically derive clinical information. The aim of this study is to investigate the accuracy of a deep learning (DL) approach for automatic segmentation of cardiac structures from CMR images characterized by magnetic susceptibility artifact in patient with cardiac implanted electronic devices (CIED).

**Methods:**

In this retrospective study, 230 patients (100 with CIED) who underwent clinically indicated CMR were used to developed and test a DL model. A novel convolutional neural network was proposed to extract the left ventricle (LV) and right (RV) ventricle endocardium and LV epicardium. In order to perform a successful segmentation, it is important the network learns to identify salient image regions even during local magnetic field inhomogeneities. The proposed network takes advantage from a spatial attention module to selectively process the most relevant information and focus on the structures of interest. To improve segmentation, especially for images with artifacts, multiple loss functions were minimized in unison. Segmentation results were assessed against manual tracings and commercial CMR analysis software cvi^42^(Circle Cardiovascular Imaging, Calgary, Alberta, Canada). An external dataset of 56 patients with CIED was used to assess model generalizability.

**Results:**

In the internal datasets, on image with artifacts, the median Dice coefficients for end-diastolic LV cavity, LV myocardium and RV cavity, were 0.93, 0.77 and 0.87 and 0.91, 0.82, and 0.83 in end-systole, respectively. The proposed method reached higher segmentation accuracy than commercial software, with performance comparable to expert inter-observer variability (bias ± 95%LoA): LVEF 1 ± 8% vs 3 ± 9%, RVEF − 2 ± 15% vs 3 ± 21%. In the external cohort, EF well correlated with manual tracing (intraclass correlation coefficient: LVEF 0.98, RVEF 0.93). The automatic approach was significant faster than manual segmentation in providing cardiac parameters (approximately 1.5 s vs 450 s).

**Conclusions:**

Experimental results show that the proposed method reached promising performance in cardiac segmentation from CMR images with susceptibility artifacts and alleviates time consuming expert physician contour segmentation.

**Supplementary Information:**

The online version contains supplementary material available at 10.1186/s12968-022-00899-5.

## Introduction

Cardiovascular magnetic resonance (CMR) represents the gold standard imaging technique for a comprehensive analysis of cardiac structure and function through the assessment of left ventricular (LV) and right ventricular (RV) volumes, LV myocardial mass (LVM), wall thickness, and ejection fraction (EF) [1]. To obtain these parameters, an accurate delineation of the LV and RV endocardium and epicardium = is required, which is operator experience-dependent. To reduce the pitfalls from manual delineation, accurate algorithms for automatic contour extraction (i.e., segmentation) are emerging in order to reduce inter/intra-observer variability and time of analysis.

Developing automatic algorithms for accurate cardiac chamber segmentation represents a challenging task, especially when considering the geometrical and dynamic changes of the heart across phases and pathologies, the presence of trabeculae and papillary muscles and the fuzzy boundaries of the ventricular cavities [2–4]. In addition, CMR suffers from noise and artifacts due to the nature of signal detection and field inhomogeneity which affects the spatial encoding of the signal [5].

Recently, with the increasing use of pacemakers and implanted cardioverter-defibrillators (ICDs) [6], metallic susceptibility artifacts are causing distortion of the magnetic field, with consequent degradation of the image quality in those patients [7].

Among automatic segmentation methods, deep learning (DL) has drawn the attention of the medical-image analysis community [8]. The common idea behind DL is to use an artificial neural network that simulates human brain and learns discriminative features from images; thus, benefiting from increased availability of medical images for training, DL-based methods have gradually emerged, outperforming previous state-of-the-art approaches for the detection and segmentation of cardiac regions [8–11].

Recently, DL is gaining attention in the field of noise and artifact reduction in CMR [10, 12–14]. However, although many promising algorithms were developed, several limitations in overcoming artifacts using DL remain. Limited datasets are often a common problem in medical image analysis. Despite datasets generated simulating artifacts are generally used, discrepancy between simulated and acquired datasets exists. Furthermore, it is challenging to prepare the ground truth datasets without artifact images for proper clinical evaluation, limiting the development of DL algorithms in clinical practice.

One of the main challenges in automatic cardiac structure segmentation from images with susceptibility artifacts is how to automatically locate the anatomical structures due to image distortion. Moreover, detecting cardiac structures is even more difficult because of the considerable variations in shape, size and position of the cardiac chambers among patients. Furthermore, it is problematic to determine the fuzzy boundaries between structures because cardiac implanted electronic devices (CIEDs) causes severe distortion in images.

To address this problem, we propose a novel DL approach based on convolution neural network (CNN) with a spatial attention mechanism which could help focus on relevant regions for automatic segmentation of LV, RV and LV mass (LVM) on short-axis (SAx) cine CMR images even when affected by susceptibility artifacts.

## Methods

### Study population

A multicenter retrospective study in a cohort of consecutive patients who were referred for CMR was conducted. To develop and validate the proposed DL approach, two selected datasets were collected: internal and external. The internal dataset included SAx cine CMR images obtained from 230 patients at IRCCS Centro Cardiologico Monzino (Milan, Italy) between May 2017 and December 2021.

The inclusion criteria were patients who underwent routine clinical CMR from various clinical indications; most of them are the evaluation of cardiomyopathy (26%), ischemic heart disease (41%), or ventricular arrhythmia (33%) (Table 1). Exclusion criteria were contraindications to CMR.

**Table 1:** Patient characteristics

	All patients (N = 230)	Patients with CIED (n = 100)	Patients without CIED (n = 130)	p value
Age, years	56 ± 17	60 ± 17	54 ± 17	0.014
Female	68 (30%)	17 (17%)	51 (39%)	0.004
Body surface area, m^2^	1.9 ± 0.2	1.9 ± 0.2	1.9 ± 0.2	0.167
Cardiac rhythm				
Sinus rhythm	211 (92%)	85 (85%)	126 (97%)	0.146
Atrial fibrillation	19 (8%)	15 (15%)	4 (3%)	
Main clinical indication for CIED implantation				
AV block or symptomatic bradycardic	6 (3%)	6 (6%)	0 (0%)	–
Cardiac arrest or sustained ventricular tachycardiac	76 (33%)	76 (76%)	0 (0%)	–
LVEF ≤ 35%	23 (10%)	23 (23%)	0 (0%)	–
Main clinical indication for CMR				
Coronary artery disease	72 (31%)	25 (25%)	47 (36%)	0.515
Ventricular arrhythmia	75 (33%)	35 (35%)	40 (31%)	
Dilated cardiomyopathy	41 (18%)	21 (21%)	20 (15%)	
Hypertrophic cardiomyopathy	18 (8%)	8 (8%)	10 (8%)	
Acute myocardial infarction	24 (10%)	11 (11%)	13 (10%)	
LVEDV index (mL/m^2^)	90 (73–108)	101 (81–124)	85 (71–100)	< 0.001
LVESV index (mL/m^2^)	42 (31–59)	50 (41–86)	38 (28–50)	< 0.001
LVEF (%)	51 ± 9	45 ± 14	54 ± 11	< 0.001
RVEDV index (mL/m^2^)	76 (66–91)	79 (67–93)	75 (64–88)	0.203
RVESV index (mL/m^2^)	34 (26–42)	37 (27–49)	33 (25–40)	0.006
RVEF (%)	55 ± 9	52 ± 10	57 ± 7	< 0.001
Permanent pacemaker	16 (7%)	16 (16%)	–	–
ICDs	84 (37%)	84 (84%)	–	–

Values are mean ± SD, median (interquartile range), or n (%)

*CIED* cardiac implanted electronic devices, *AV* atrioventricular, *LV* left ventricular, *RV* right ventricular, *EDV* end diastolic volume, *ESV* end systolic volume, *EF* ejection fraction, *ICDs* implanted cardioverter-defibrillators

CMR studies were performed using a 1.5 T system (Discovery MR450, General Electric Healthcare, Chicago, Illinois, USA) equipped with a 32-channel cardiac coil. Breath-hold balanced steady-state free-precession cine acquisitions were performed in vertical and horizontal long-axis orientations and in SAx orientations. A stack of SAx slices encompassing both ventricles from base to apex was used for biventricular volumes and LVM assessment. All images (512 × 512 pixels) were acquired with in-plane resolution ranging from 1.2 to 2.0 mm, echo/repetition time (TE/TR) echo time 1.6/3.7 ms, 80–85° flip angle, bandwidth 488 kHz, slice thickness of 8 mm, no interslice gap and a field of view ranging from 300 to 360 mm. For each patient, images were acquired with 30 phases/cardiac cycle. A SAx image stack typically consists of 10 image slices. The study protocol was approved by the institutional review board, which waved informed consent for this retrospective study.

Patients were subdivided into two subgroups, considering the presence (group 1, n = 100) or absence (group 2, n = 130) of a CIED (either pacemaker or ICD). This last group of patients, characterized by images without artifacts, was included to achieve an adequate training sample size to reduce overfitting and to generalize the network’s ability in both artifacts and artifacts-free images. The baseline characteristics of the patients’ cohort are reported in Table 1.

An external testing dataset for 56 patients with CIED acquired at Fondazione Toscana Gabriele Monasterio (Pisa, Italy) from 2016 to 2022 was used to test the generalizability of the developed model. The external testing dataset consisted of SAx cine CMR images (256 × 256 pixels) acquired during breath hold, including both ventricles from base to apex, using a 1.5 T CMR scanner (Signa Excite, General Electric Healthcare; or Signa Artist, General Electric Healthcare). The following acquisition parameters were applied: TR = 3.1–3.9 ms, TE = 1.5–1.8 ms, flip angle = 45°–60°, 30 cardiac phases, pixel resolution = 1.3–1.6 mm, slice thickness = 8 mm, no interslice gap and bandwidth = 62.5–200 kHz.

### CMR analysis and image preparation

The gold standard was represented by manual tracing of the contours of the LV and RV cavity and of LVM, performed by one clinical reader (European Association for Cardiovascular Imaging (EACVI) Level III CMR certified reader) on the stack of cine SAx CMR frames corresponding to the end-diastolic (ED) and end-systolic (ES) phases, using cvi^42^ (version 5.11, Circle Cardiovascular Imaging Inc., Calgary, Alberta, Canada). The ED and ES frames were respectively chosen as the images with the largest and the smallest LV blood volume at the mid-ventricular level. In both phases, the most basal slice for the LV was selected when at least 50% of the LV blood pool was surrounded by myocardium. The LV papillary muscles and trabeculae were included as part of LV and RV cavities, in agreement with the guidelines of the Society for Cardiovascular Magnetic Resonance (SCMR) [15]. For the RV, the slices below the pulmonary valve were included. During the tracing process, in both ED and ES, the contours were adjusted so that LVM would result as similar as possible. As well, the LV and RV stroke volumes were checked to ensure their similarity. In case of doubt in the tracings, the contours were reviewed and corrected based on the second opinion of an additional expert. A total number of 4198 and 893 CMR images that included both ED and ES slices were used for the internal and external dataset, respectively.

CMR images were exported in DICOM format, rotated to the right- anterior-head reference frame, and cropped and resized to 192 × 192 pixels to reduce computational and memory requirements. Image intensity was normalized in the [0,1] range.

From the available internal dataset, CMR studies were randomly split (patient-wise) into training (group 1: 70%, group 2: 70%) and validation (group 1: 15%, group 2: 10%) for determining the optimal model parameters. The remaining CMR studies (group 1: 15%, group 2: 20%) were used for testing the models.

### Segmentation network

**Fig. 1:**
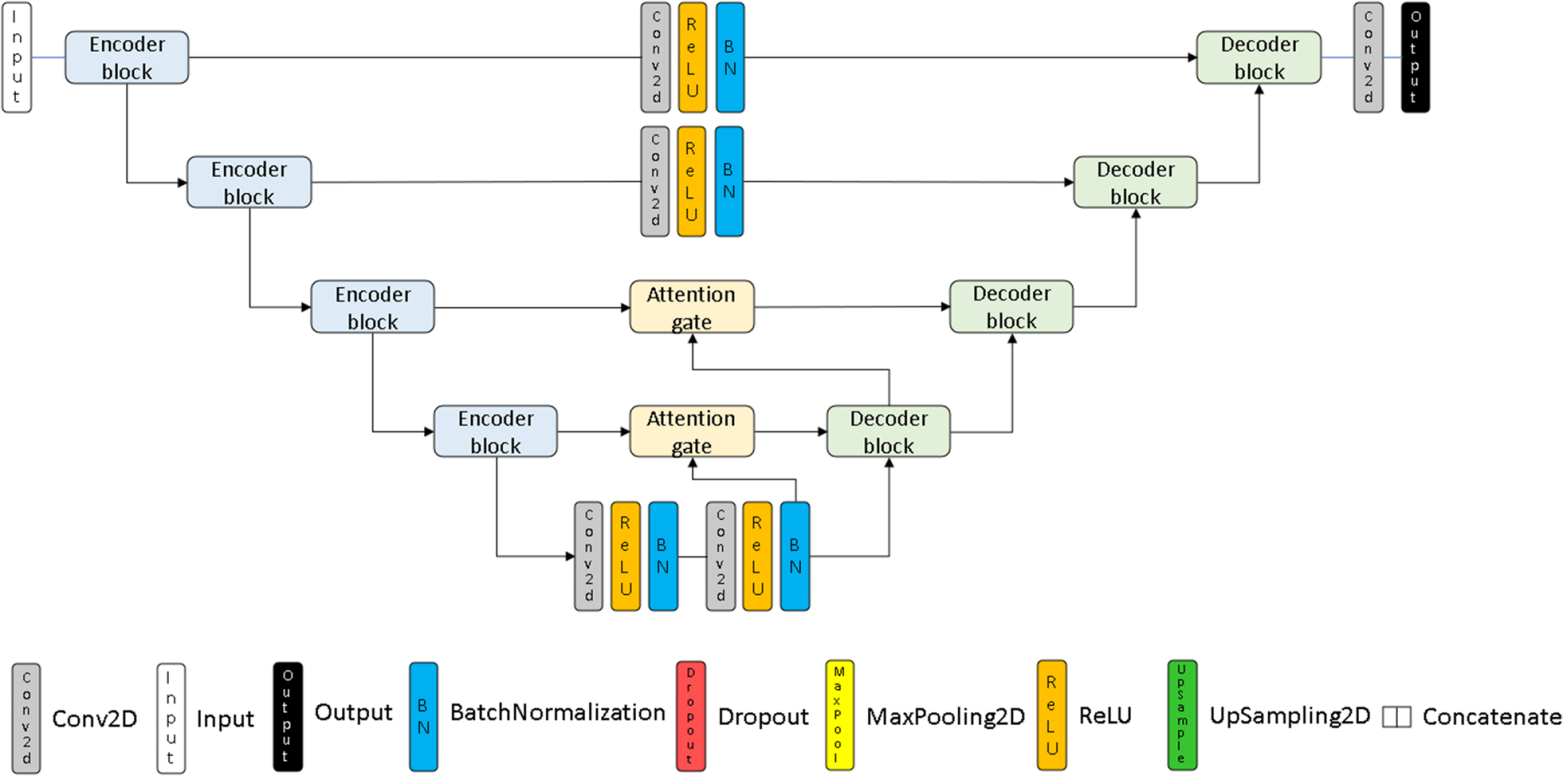
Convolutional neural network architecture

In order to perform the segmentation of LV, RV and LVM in images characterized by metallic susceptibility artifacts, a DL technique based on CNNs was proposed, in which a spatial attention module capable to identify salient image regions even in the presence of artifacts was introduced. CNNs are neural networks designed to automatically learn spatial hierarchies of features, from low- to high-level patterns [1, 8]. The proposed CNN leverages the U-Net model architecture, a very successful architecture for semantic segmentation in medical image analysis that enables learning from relatively small number of training samples [4, 10]. Typically, U-Net includes an encoder-decoder structured to extract contextual information and to enable precise segmentation and skip connections that combines high-resolution local features with low-resolution global features. Figure 1 depicts the proposed network architecture. The encoder (Additional file 1: Figure S1) is characterized by a series of convolutional and pooling layers that doubles the size of the feature map while reducing the number of channels by half. Each convolution uses a 3 × 3 kernel and it is followed by batch normalization and ReLU activation function [16, 17]. The decoder (Additional file 1: Figure S2) recovers the spatial information back to the image space through a series of upsampling and convolution operations, thus increasing the output resolution. The decoder connects the upsampling features with those of the corresponding portion of the encoder through the skip connections. The resulting features map is convoluted to match the same number of channels of the corresponding portion of the encoder.

Unlike U-Net, due to the semantic gaps between low-level encoder feature and the corresponding high-level decoder features, the original skip connection architecture was modified adding attention gates (AGs) in the bottom two-layer levels and convolutional layer with 1 × 1 kernel in the top two-layer levels [18]. To improve segmentation accuracy, reducing the number of false-positive prediction in structures that present large variability, such as for ventricular chambers, AGs emphasize salient image regions, preserving relevant activations to the specific task and propagating them to the decoding stage [18, 19]. Therefore, AGs generate a spatial attention map, focusing on the informative parts and progressively suppressing feature responses in irrelevant background regions. This allows the network to be more robust to noisy input, as in the case of CMR images with susceptibility artifacts. The structure of an AG is shown in Additional file 1: Figure S3. At the final layer, a 1 × 1 convolution and a Softmax activation function are used to get the output segmentation map.

To improve segmentation accuracy, especially in more complicated examples (such as images with susceptibility artifacts), the network was trained with a combination of weighed cross-entropy and Focal Tversky loss function [4, 19, 20]. The cross-entropy loss has the advantage of speeding up the learning process at the beginning of the training and is able to deal with the label unbalance typical of medical images analysis, while the Focal Tversky loss helps in improving the recall rate, thus leading to a better balance between precision and recall [18–20]. In addition, Focal Tversky loss increases the degree of focusing on more critical examples by down-weighting the easy ones. To verify the effectiveness of each component, an ablation study was performed. (1) We investigate the impact of the AG module, replacing it with standard convolutional layer with 1 × 1 kernel; (2) We also analyzed the impact of the Focal Tversky loss. For the ablation study, we use the internal testing datasets for all our experiments.

Initial weight values were extracted from a normal distribution [21]. To speed up the learning efficiency and reduce the number of epochs, the model was optimized using Adam optimizer with a learning rate of 1e-4 and batch size of 4. During training, the learning rate was set to decrease to 0.04 after each epoch, where an epoch is defined as the iteration over all training images. The maximum training epoch limit was set to 60. After each epoch, validation dice similarity coefficient (DSC) was evaluated, and the model with the highest validation DSC was selected as final model. The DSC is defined as:

1 $$DSC = \frac{2\left|X\cap Y\right|}{\left|X\right|+\left|Y\right|}$$

and represents a measure of overlap between the predicted volume and the corresponding reference volume. The DSC index gives a value between 0 (no overlap) and 1 (full overlap). To improve the robustness and generalization capabilities of the model, minimizing overfitting during training, data augmentation was applied on-the-fly with a combination of random rotations in [− 30°, 30°] range and gamma correction. The implementation was based on Python and Tensorflow (version 2.1).

### Evaluation and statistical analysis

To evaluate the performance of the proposed method, four commonly used metrics were used: DSC, Hausdorff distance (HD), Recall (Rec) and Precision (Prec). The HD measures the local maximum distance between the predicted and the manual segmentation. Rec and Prec are defined as:

2 $$Rec = \frac{TP}{TP+FN}$$

3 $$Prec = \frac{TP}{TP+FP}$$

where true positive (TP) and true negative (TN) are the number of correctly predicted voxels belonging to the target class and the background class, respectively, and false positive (FP) and false negative (FN) are the number of misclassified voxels as the object and background respectively.

Rec and Prec are in range [0,1], where higher values indicate better performance.

Based on the results of the pixel classification for each patient, several clinical parameters, namely the ED and ES volumes (LVEDV, LVESV, and RVEDV, RVESV expressed in mL for the LV and RV, respectively), the ejection fractions (LVEF and RVEF expressed in percent for the LV and RV, respectively), and the myocardium mass (LVM ED and LVM ES expressed in g and calculated at ED and ES, respectively), were computed and compared against the corresponding values obtained manually using the commercial software by intra‐class correlation coefficient (ICC) and Bland–Altman analyses. Good reproducibility was indicated by an ICC > 0.75 between measurements.

To assess the benefit of the developed methodology in reducing inter-observer variability, an additional expert cardiologist (O2, EACVI Level III CMR certified reader) independently manually annotated the images with artifacts of the test subjects and the inter-observer variability between the manual segmentations of different experts (O1, O2) was evaluated. In addition, difference in clinical parameters was computed between the two observers, and compared with the automated ventricular boundary detection results obtained by (1) the CNN and (2) cvi^42^ contours versus the manual segmentation. Both CNN and cvi^42^ adopted an automatic DL contour tracing of the LV (endocardial and epicardial) and RV (endocardium) borders on manually selected ED and ES phases, in order to ensure that annotations covered the same time frames.

The time required to obtain the volume segmentation (ED and ES phases) using the proposed CNN and by the expert physician by manual tracing was also reported.

To evaluate the level of artifacts, image quality was determined by an expert observer. For each examination, image quality was scored ranging from 0 = reduced diagnostic quality with many artifacts to 1 = diagnostic quality with many artifacts, 2 = good diagnostic quality with some artifacts, 3 = optimal diagnostic quality. Criteria involved overall image quality concerning diagnostic value and artifacts.

Continuous data are presented as mean ± standard deviation (SD) or median (interquartile range) and categorical variables as absolute frequencies (percentages), as appropriate. Method comparisons were analyzed using Mann–Whitney test. Differences between subgroups (i.e., group 1 and group 2) were assessed using an unpaired Student’s t-test for continuous variables (and the Welch’s corrected version, as appropriate) or the Mann–Whitney U test, whilst an χ^2^ test was applied for categorical data. The results were considered significant with p values < 0.05. Statistical analysis was conducted using SPSS (version 27.0, SPSS Inc, Statistical Package for the Social Sciences, International Business Machines, Inc., Armonk, New York, USA).

## Results

### Performance on artifact-free images

Table 2 compares the model-predicted segmentation labels with the ground truth segmentation labels on artifact-free images in terms of DSC, HD, Rec and Prec for each ventricular structure (LV and RV endocardium and LV myocardium) in the ED and ES frames. Specifically, the median DSC for LV, RV and LVM, was 0.97, 0.95 and 0.87 in ED and 0.95, 0.91, and 0.90 in ES, respectively. The median DSC, HD, Rec and Prec values for both LV and RV at ED tended to be slightly better than at ES. The median HD varied between 2.3 and 4.4 mm.

**Table 2:** Segmentation performance of the CNN on artifact-free images

LV ED				LV ES			
DSC	HD (mm)	Recall	Precision	DSC	HD (mm)	Recall	Precision
0.97 (0.96–0.97)	2.7 (2.2–4.5)	0.96 (0.94–0.96)	0.99 (0.98–0.99)	0.95 (0.94–0.95)	2.8 (2.3–4.2)	0.95 (0.92–0.97)	0.96 (0.91–0.98)

RV ED				RV ES			
DSC	HD (mm)	Recall	Precision	DSC	HD (mm)	Recall	Precision
0.95 (0.94–0.96)	3.0 (2.3–4.5)	0.95 (0.93–0.96)	0.95 (0.94–0.96)	0.91 (0.87–0.92)	4.4 (3.2–8.0)	0.91 (0.89–0.94)	0.91 (0.84–0.94)

LVM ED				LVM ES			
DSC	HD (mm)	Recall	Precision	DSC	HD (mm)	Recall	Precision
0.87 (0.85–0.89)	2.3 (1.9–3.2)	0.93 (0.88–0.94)	0.82 (0.79–0.87)	0.90 (0.87–0.90)	2.7 (2.4–3.6)	0.93 (0.89–0.95)	0.86 (0.84–0.91)

Values are median (interquartile range)

*ED* end-diastolic, *ES* end-systolic, *DSC* dice coefficient, *HD* Hausdorff distance, *LV* left ventricular, *RV* right ventricular

Additional file 1: Figure S4 and Table S1 shows the clinical parameters calculated using CNN automated segmentation compared to the manual gold standard on artifact-free images. For both LV and RV volumes, high ICC (> 0.97) was obtained; also, the LVEF and RVEF resulted in strong correlation (> 0.94), near zero bias and narrow confidence intervals. LVM demonstrated good ICC (i.e., 0.75 in ED and 0.88 in ES), reasonable bias and wider limits of agreement. These results are in agreement with the results reported in Table 2, where uncertainties in pixel classification affected clinical parameter estimations.

**Fig. 2:**
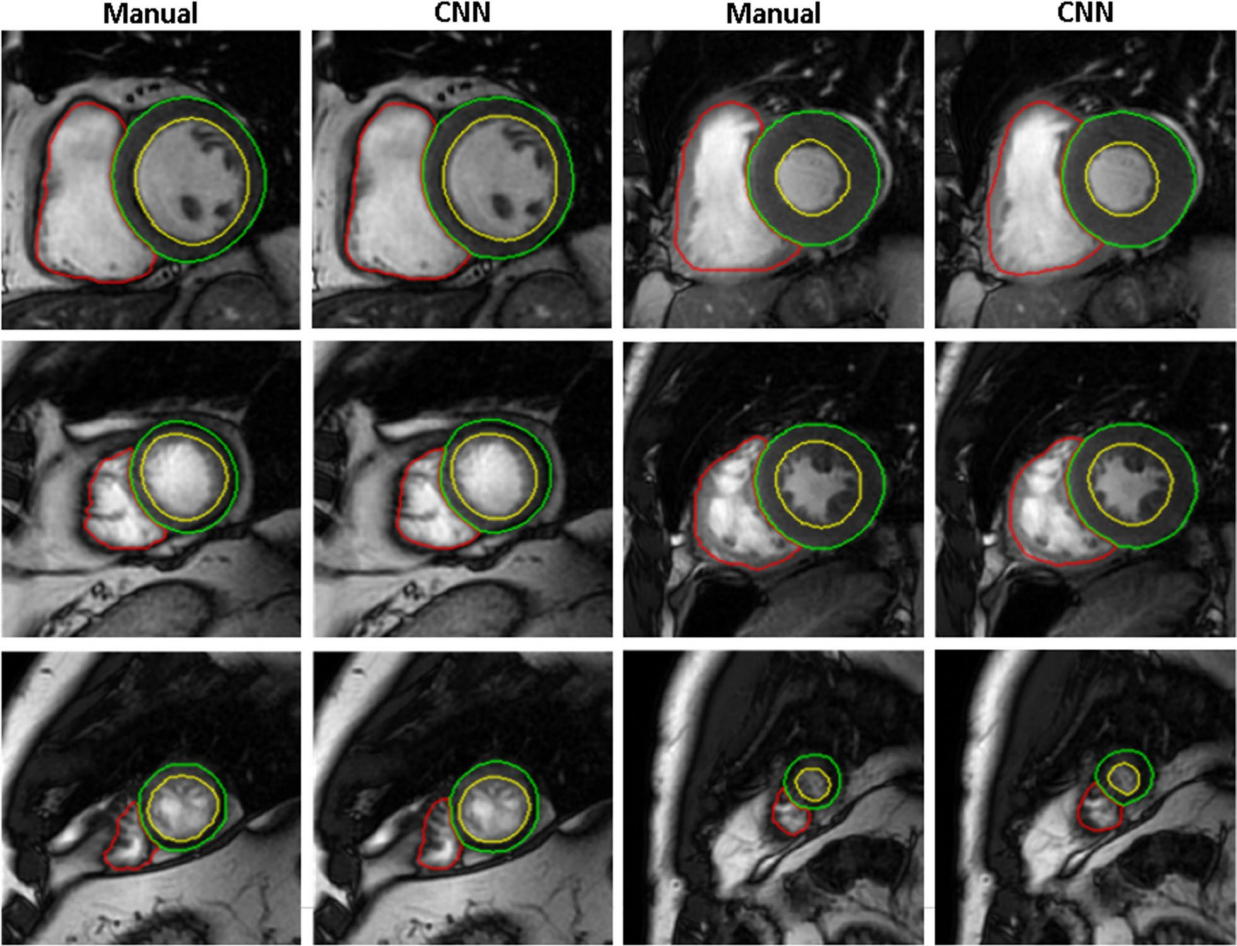
Example of segmentation results obtained using the proposed convolutional neural network (CNN) compared to the manually traced gold standard on cases without artifacts (LV blood-pool: yellow; RV blood-pool: red; myocardium: green)

Figure 2 shows examples of the model segmentation at different slice locations for patients without CIED, paired with the corresponding gold standard manual tracings. Qualitatively the segmentation yielded convincing results, demonstrating a good agreement with the manual segmentation.

### Performance on susceptibility artifacts images

**Fig. 3:**
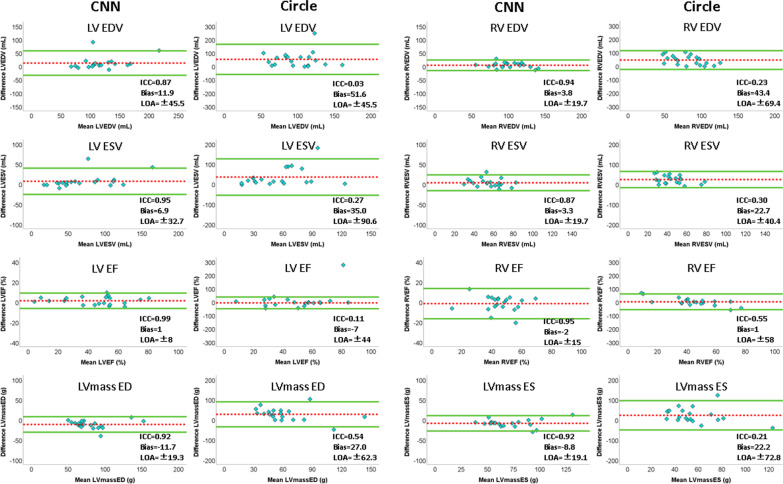
Results of correlation and Bland–Altman analysis using the developed CNN and the commercial cardiovascular magnetic resonance (CMR) software (cvi^42^, Circle Cardiovascular Imaging, Calgary, Alberta, Canada) versus manual measurement on cases with artifacts. Dashed line = bias; solid line = ± 2 standard deviations

In the internal testing dataset, comparing the performance of the proposed CNN and of cvi^42^ software compared to the manual gold standard for the images with magnetic susceptibility artifacts, and considering interobserver variability, the evaluation measures (DSC, HD, Rec and Prec in Table 3) showed a better performance of the proposed CNN compared to the commercial software, with similar values when comparing the two observers’ tracings. Overall, the CNN performed well on images with artifacts, with a median DSC for LV, RV and LVM significantly higher than that of cvi^42^ software. These results are further corroborated by the higher ICC values with the gold standard in the EF obtained with the CNN segmentation than those obtained with cvi^42^ software (Fig. 3), in particular for the EF (LVEF ICC: 0.99 vs 0.11; RVEF ICC: 0.954 vs 0.55). Except for the LV ES volume, the median DSC values of the CNN segmentation were all in the range of the inter-observer variability (Table 3). As for the HD scores, the difference between automated and ground truth segmentation was smaller or slightly above (< 3 mm) the inter-observer results. Also, comparing the results of ICC and Bland–Altman analysis of the clinical measures between CNN and the gold standard (Fig. 3), as well as between two observers, it is possible to appreciate how the CNN resulted in smaller bias for LVEF and RVEF compared to human variability (Additional file 1: Table S2). By contrast, the bias of the CNN was larger compared to that of expert variability for both LV and RV volumes, as well as for LVM, although the CNN resulted in a strong ICC and narrow confidence intervals for both RV volume and LVM.

**Table 3:** Internal validation: segmentation performance on images with artifacts

	LV ED				LV ES			
	DSC	HD (mm)	Recall	Precision	DSC	HD (mm)	Recall	Precision
CNN vs GT	0.93 (0.91–0.95)⁎	7.8 (4.7–11.8)	0.92 (0.86–0.94)⁎	0.95 (0.93–0.98)⁎	0.91 (0.84–0.93)⁎	6.0 (4.6–9.5)⁎	0.88 (0.84–0.94)⁎	0.94 (0.83–0.95)⁎
Circle vs GT	0.43 (0.25–0.86)†	10.6 (2.7–20.0)	0.34 (0.18–0.80)†	0.61 (0.41–0.90)†	0.63 (0.14–0.83)†	11.1 (4.1–20.3)†	0.56 (0.08–0.77)†	0.74 (0.30–0.88)†
O1 vs O2	0.93 (0.82–0.95)	5.2 (3.5–10.2)	0.91 (0.78–0.97)	0.93 (0.88–0.95)	0.92 (0.85–0.96)	6.1 (3.4–8.1)	0.94 (0.85–0.97)	0.91 (0.84–0.93)

	RV ED				RV ES			
	DSC	HD (mm)	Recall	Precision	DSC	HD (mm)	Recall	Precision
CNN vs GT	0.87 (0.84–0.91)⁎	10.5 (6.8–14.3)⁎	0.85 (0.81–0.90)⁎	0.91 (0.86–0.93)⁎†	0.83 (0.73–0.90)⁎	9.3 (6.7–13.9)⁎†	0.81 (0.70–0.89)⁎	0.88 (0.80–0.92)⁎†
Circle vs GT	0.59 (0.21–0.78)†	25.5 (15.7–47.3)	0.45 (0.14–0.72)†	0.74 (0.42–0.85)†	0.50 (0.19–0.82)†	21.4 (7.9–38.2)	0.43 (0.13–0.78)†	0.69 (0.31–0.86)
O1 vs O2	0.85 (0.70–0.90)	13.0 (6.5–20.6)	0.86 (0.70–0.95)	0.82 (0.73–0.86)	0.76 (0.70–0.85)	14.1 (9.5–24.3)	0.85 (0.65–0.93)	0.72 (0.65–0.79)

	LVM ED				LVM ES			
	DSC	HD (mm)	Recall	Precision	DSC	HD (mm)	Recall	Precision
CNN vs GT	0.77 (0.71–0.82)⁎†	6.5 (5.2–9.0)⁎	0.82 (0.79–0.87)⁎†	0.70 (0.67–0.79)⁎†	0.82 (0.73–0.84)⁎	7.1 (5.1–11.4)	0.84 (0.76–0.91)⁎†	0.75 (0.70–0.81)⁎
Circle vs GT	0.41 (0.15–0.70)†	14.8 (6.8–30.4)	0.31 (0.11–0.68)†	0.50 (0.29–0.74)†	0.59 (0.10–0.79)†	10.7 (5.5–25.4)†	0.51 (0.07–0.79)†	0.59 (0.26–0.83)†
O1 vs O2	0.71 (0.62–0.78)	6.4 (3.7–11.8)	0.75 (0.68–0.79)	0.70 (0.61–0.80)	0.76 (0.66–0.81)	8.2 (4.3–9.9)	0.72 (0.62–0.85)	0.76 (0.71–0.84)

Values are reported as median (interquartile range)

Segmentation performance on images with artifacts for the proposed CNN and for the commercial software (Circle) compared to the manual gold standard (GT). Also, the results of the comparison between two observers (O1 vs O2) is reported

Abbreviations as in Table 2. ⁎p < 0.05 CNN vs. Circle; †p < 0.05 vs. inter-observer variability

**Fig. 4:**
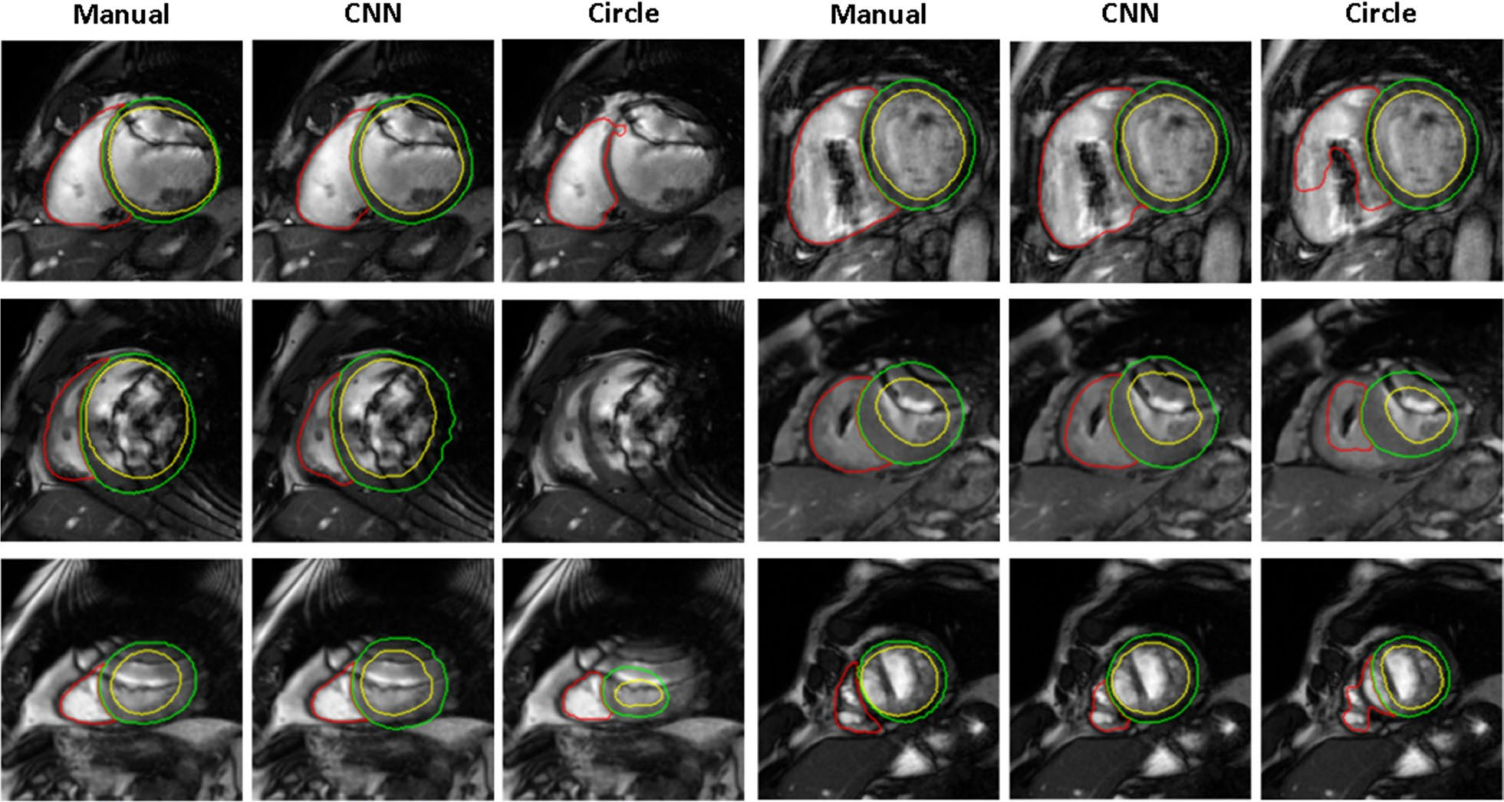
Example of segmentation results obtained from short-axis CMR images affected by susceptibility artifacts using the developed CNN vs commercial CMR software (cvi^42^) compared to the manually traced gold standard at different slice locations (LV blood-pool: yellow; RV blood-pool: red; myocardium: green)

Figure 4 shows examples on images with susceptibility artifacts from the internal testing dataset, including the automated segmentation results obtained both with cvi^42^ software and with the proposed method. It is possible to appreciate how all the contours were properly depicted using the proposed CNN, with results comparable to the manual tracings, while the commercial software resulted in suboptimal segmentation of the LV and RV endocardium and LV epicardium in the presence of susceptibility artifacts caused by CIED affecting the quality of the image.

**Fig. 5:**
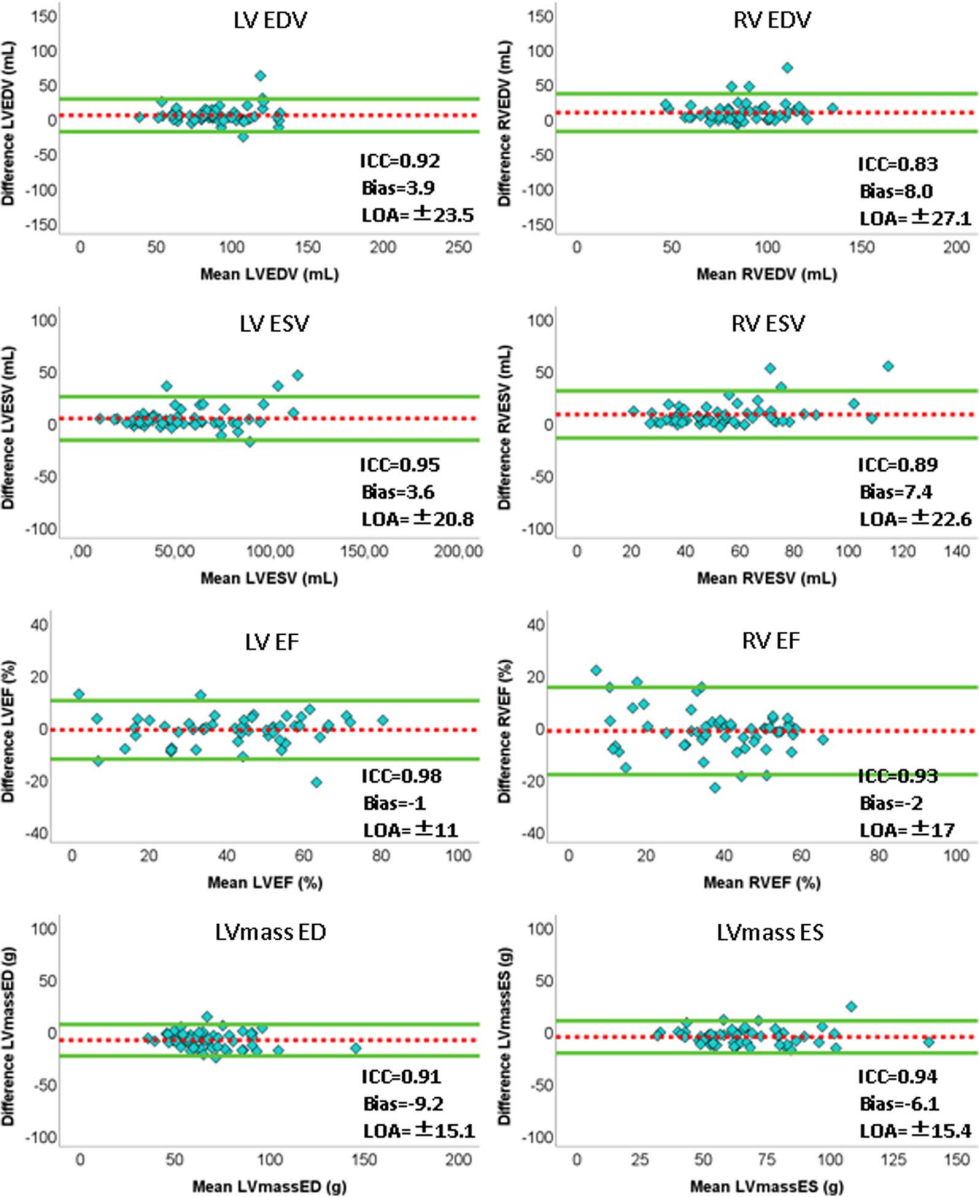
Results of correlation and Bland–Altman analysis of automated measurements versus manual measurement on the external testing dataset. Dashed line = bias; solid line = ± 2 standard deviations

As regards segmentation performance on the external testing dataset, the proposed CNN showed similar or slightly better performance compared to the internal dataset (see Table 4). Most of the predicted contours have a median DSC index equal or over 0.8, especially on both LV and RV. Regarding the volumes, by looking at Fig. 5 and Additional file 1: Table S3, it can be seen that for LV volume and LVM the CNN resulted in a strong ICC (> 0.9) and narrow limits of agreement. For RV volume, still a good ICC was reported (> 0.8) with a wider (but still acceptable) limits of agreement. The ICC corresponding to LVEF and RVEF were 0.98 and 0.93 respectively, thus indicating that the proposed method correlates highly with the clinical expert manual tracings.

**Table 4:** External validation: segmentation performance on images with artifacts

	LV ED				LV ES			
	DSC	HD (mm)	Recall	Precision	DSC	HD (mm)	Recall	Precision
CNN vs GT	0.94 (0.91–0.96)	5.2 (3.2–8.5)	0.94 (0.88–0.97)	0.96 (0.94–0.97)	0.92 (0.94–0.94)	4.8 (3.2–8.5)	0.92 (0.82–0.95)	0.94 (0.91–0.97)

	RV ED				RV ES			
	DSC	HD (mm)	Recall	Precision	DSC	HD (mm)	Recall	Precision
CNN vs GT	0.90 (0.84–0.92)	8.2 (5.8–12.9)	0.87 (0.80–0.93)	0.92 (0.89–0.94)	0.88 (0.80–0.91)	8.2 (4.8–13.1)	0.85 (0.77–0.90)	0.92 (0.88–0.94)

	LVM ED				LVM ES			
	DSC	HD (mm)	Recall	Precision	DSC	HD (mm)	Recall	Precision
CNN vs GT	0.79 (0.75–0.83)	5.0 (3.2–6.5)	0.87 (0.81–0.91)	0.75 (0.68–0.79)	0.83 (0.78–0.87)	4.4 (3.5–6.8)	0.88 (0.83–0.92)	0.79 (0.71–0.85)

Values are reported as median (interquartile range)

Segmentation performance on images with artifacts for the proposed CNN compared to the manual gold standard (GT)

Abbreviations as in Table 2

The mean time required for one volume segmentation and EF measurements on images with artifacts for the CNN and the expert physician was approximately 1.5 s and 450 s, respectively.

Image quality was evaluated by an experienced observed. The internal testing dataset had a mean image quality score of 1.2 (± 0.8), while the external testing dataset reported a slightly higher diagnostic quality compared to the cine images of the internal dataset (1.6 ± 1.1).

### Ablation study

From the ablation analysis, the CNN with the AG module leads to better segmentation results, suggesting as this attention mechanism may help to highlight salient features that are later merged through skip connections. Specifically, for LV, RV and LVM, respectively, the network without AGs achieved a mean Dice of 0.92, 0.83 and 0.76 versus 0.92, 0.85 and 0.80 of the proposed CNN. Even compared to the network without the Focal Tversky loss, the proposed network reached slightly better performance for both LV (0.92 vs 0.91), RV (0.85 vs 0.83) and LVM (0.80 vs 0.78).

## Discussion

In this multicenter study, a novel deep CNN for automatic segmentation of cardiac structures in CMR images affected by magnetic susceptibility artifacts was developed and tested. The developed method was able to obtain accurate image segmentation, matching expert physician performance and clinical measurement accuracy in both artifacts and artifacts-free cine CMR images. This allows for automatic and faster segmentation of cardiac anatomy than manual tracing.

In the last few decades, CMR has been largely adopted in diagnostic strategies, with an increase in the number of CMR scans [22]. With the development of CMR-conditional CIEDs, performing CMR scans in patients with a pacemaker or ICD has become part of daily clinical routine [23]. Several studies have demonstrated safety of CMR for patients with CIEDs [7, 24, 25]. Approximately 30% of patients with CIEDs are expected to undergo CMR analysis within a period of 4 years of implantation, with one third of them requiring more than one scan [22]. As CIEDs cause susceptibility artifacts in the images, thus leading to a longer processing time for their analysis, the proposed solution tackles this problem, with a performance comparable to that obtained with manual tracing. To the best of our knowledge, this is the first DL method developed and applied to the segmentation of cardiac structures in CMR images obtained from patients with CIEDs.

Pacemakers and other CIEDs lead to susceptibility artifacts which occurred in different image regions, most pronounced in the mid antero-septal, infero-septal and apical-septal myocardial segments [25]. Furthermore, the size of the area affected by artifacts might be significantly different among patients, further increasing their appearance variability, and thus making image segmentation challenging even for DL-based algorithms.

To deal with this problem, using an attention map might help the network to focus more on relevant information. Attention represents an important aspect of the human perception. One unique characteristic of the human visual system is the ability to selectively process the whole scene in order to capture and focus on relevant aspects of the visual scene [26]. Several recent attempts have been made to mimic the concept of attention into CNN to improve the model performance in various visual tasks [18, 19, 27, 28]. Suppressing activations from irrelevant parts of the image, the network can highly benefit for organ identification and localization, even in the presence of noisy images, following the same methodology of an expert physician by identifying the structure of interest and then focusing on it for a detailed analysis.

Based on the results from the ablation study, the AG module and the Focal Tversky loss contribute to the performance improvement as they are specifically dedicated to the attention. Indeed, spatial attention mechanism can enhance important features, suppressing unimportant ones, thus leading to improved network performance. In addition, the Focal Tversky loss might help the network to focus on hard cases, alleviating the performance degradation caused by magnetic susceptibility artifacts.

By carefully comparing the difference between manual and automated contours, the RV tracing proved to be more tedious than LV segmentation, as demonstrated by the lower DSC values and higher HD. Indeed, due to the irregular cavity and the complex crescent-shaped structure, the accurate segmentation of the RV is affected. Furthermore, a higher similarity of the signal intensity with the surrounding structures, makes RV contour detection more complicated than the LV, thus limiting the accuracy of the segmentation process. This is also corroborated by the higher inter-observer variability of the RV structure compared to the LV. Also, by carefully comparing the segmentation results, it is observed as myocardium represents the most variable and tedious structure to be traced, even for experts. This is probably because accurate segmentation implies the precise delineation of both endocardium and epicardium. In addition, the LVM contours appear more irregular among different cardiac pathologies, and present fuzzy boundaries with the surrounding structures, limiting the segmentation accuracy.

Within the bounds of our study, we found that the DSC scores for the CNN were in the range of inter-observer variability, thus suggesting how DL methods could match performance of the expert physician in segmenting CMR images, not only in artifact-free images [4, 9, 29], but even when there are distortions in the magnetic field with consequent alteration in the image quality.

Although accuracy represents a relevant property of a decision support system when assisting clinicians in diagnosis, the speed of algorithm execution is also critical for improving work efficiency. Results demonstrated that the proposed CNN was 300 times faster than manual tracing in providing cardiac parameters. This would allow a reduction in the analysis time for patients with CIEDs, thus overcoming the current implications of manual delineation (i.e., time-consuming, tedious, and fatigue errors) which remains the reference standard for ventricular segmentation from CMR images with artifacts. Another important novelty is that this new proposed method allows a comprehensive analysis of both ventricles, considering that artifacts may interfere with both right and left chamber measurements.

In the search for the best strategy to multi-structure segmentation on CMR with artifacts, our results suggest that the proposed DL method is a better solution compared to the one represented by the most widespread commercial CMR software (i.e., Circle Cardiovascular Imaging), demonstrating a higher accuracy in measuring volumes and EF in patients with susceptibility artifacts, and thus reflecting the effectiveness of the developed architecture. Despite its popularity, the automated segmentation by commercial software resulted sensitive to magnetic susceptibility and image distortions, thus leading to inaccurate localization of the ventricles and major discrepancies compared to manual tracings.

### Limitations

Although our experiments have proved the effectiveness of the proposed CNN as support for cardiac clinical diagnosis, there are some limitations. First, although the utilized datasets were acquired using different CMR imaging acquisition protocols and scanner types, all CMR scanners operated at a field strength of 1.5 T. Evaluations with scanners with a higher magnetic field strength are needed. As reported in the literature [25], 3 T CMR imaging led to worsening of the susceptibility artifacts. Second, our evaluation protocol was compared on a single commercial software, while it would be desirable to expand such analysis on other CMR analysis software currently used in clinical practice. Third, although our results are encouraging with segmentation performance near to those of the expert clinicians, extending our framework with global attention mechanisms to capture the global image representation and with spatio-temporal features may further improve the performance. Finally, the model was trained only on ED and ES phases, because manual contours were provided for these two phases only; however, we expect the proposed model to be able to perform well even on the other time frames.

## Conclusion

An accurate fully automated DL model for CMR image segmentation, able to handle susceptibility artifacts caused by cardiac implantable electronic devices, was proposed and tested. Its novel CNN architecture, including attention gates to accurately locate and segment the cardiac structures, resulted in a performance in the range of the expert inter-observer variability, with high accuracy in the computed clinical parameters when compared to the ground truth. When compared to a widely used commercial CMR analysis software, the proposed network resulted in a higher automated segmentation accuracy in CMR images affected by susceptibility artifacts. The proposed method provides an end-to-end solution for CMR image segmentation of both ventricular cavities affected by susceptibility artifacts, easing and accelerating the cardiac functional analysis process.

## Supplementary Information

**Additional file 1. Table S1**. Correlations between CNN and manual gold standard on the artifacts-free images.** Table S2**. Internal validation: correlations on images with artifacts for the proposed CNN and the commercial software (Circle) in respect to the manual gold standard (GT). Also, the results relevant to interobserver variability between O1 and O2 reported for comparison.** Table S3**. External validation: correlations between CNN and manual gold standard on images with artifacts.** Figure S1**. Encoder module.** Figure S2**. Decoder module.** Figure S3**. The attention gate module.** Figure S4**. Results of correlation and Bland-Altman analysis of automated measurements versus manual measurement on cases without artifacts.

## Data Availability

The datasets generated and/or analyzed during the current study are not publicly available due institutional policies but are available from the corresponding author on reasonable request.

## Abbreviations

AG: Attention gates
CIED: Cardiac implanted electronic device
CMR: Cardiovascular magnetic resonance
CNN: Convolutional neural network
DL: Deep learning
DSC: Dice similarity coefficient
EACVI: European Association of Cardiovascular Imaging
ED: End-diastolic
EF: Ejection fraction
ES: End-systolic
HD: Hausdorff distance
ICD: Implanted cardioverter-defibrillator
LV: Left ventricle/left ventricular
LVEDV: Left ventricular end-diastolic volume
LVEF: Left ventricular ejection fraction
LVESV: Left ventricular end-systolic volume
LVM: Left ventricular myocardial mass
Prec: Precision
Rec: Recall
RV: Right ventricle/right ventricular
RVEDV: Right ventricular end-diastolic volume
RVEF: Right ventricular ejection fraction
RVESV: Right ventricular end-systolic volume
SAx: Short-axis
