# Supplementary material for: Cardiovascular magnetic resonance images with susceptibility artifacts: artificial intelligence with spatial-attention for ventricular volumes and mass assessment
Source: J Cardiovasc Magn Reson. 2022 Nov 28;24:62. doi: 10.1186/s12968-022-00899-5 (PMC9703740; doi:10.1186/s12968-022-00899-5)

Supplementary Tables

**Table 1:** Correlations between CNN and manual gold standard on the artifacts-free images.

| LVEDV | | LVESV | | RVEDV | | RVESV | |
| --- | --- | --- | --- | --- | --- | --- | --- |
| ICC | bias±2SD (mL) | ICC | bias±2SD (mL) | ICC | bias±2SD (mL) | ICC | bias±2SD (mL) |
| 0.99 | 3.3±7.0 | 0.98 | -1.4±7.7 | 0.99 | 0.2±7.6 | 0.98 | -0.9±7.2 |
| LVEF | | **RVEF** | | **LV_mass_ED** | | **LV_mass_ES** | |
| ICC | bias±2SD (%) | ICC | bias±2SD (%) | ICC | bias±2SD (g) | ICC | bias±2SD (g) |
| 0.97 | 1±6 | 0.95 | 1±6 | 0.75 | -6.2±11.7 | 0.88 | -3.3±10.3 |

*LVEDV: left ventricular end-diastolic volume; LVESV: left ventricular end-systolic volume; RVEDV: right ventricular end-diastolic volume; RVESV: right ventricular end-systolic volume; LVEF: left ventricular ejection fraction; RVEF: right ventricular ejection fraction; LVmassED: myocardial mass in diastole; LVmassES: myocardial mass in systole; ICC: intraclass correlation coefficient.*

**Table 2:** Internal validation: correlations on images with artifacts for the proposed CNN and the commercial software (Circle) in respect to the manual gold standard (GT). Also, the results relevant to interobserver variability between O1 and O2 reported for comparison.

|  | **LVEDV** | | **LVESV** | | **RVEDV** | | **RVESV** | |
| --- | --- | --- | --- | --- | --- | --- | --- | --- |
|  | ICC | bias±2SD (mL) | ICC | bias±2SD (mL) | ICC | bias±2SD (mL) | ICC | bias±2SD (mL) |
| CNN vs GT | 0.87 | 11.9±45.5 | 0.95 | 6.9±32.7 | 0.94 | 3.8±19.7 | 0.87 | 3.3±19.7 |
| Circle vs GT | 0.03 | 51.6±112.7 | 0.27 | 35.0±90.6 | 0.23 | 43.4±69.4 | 0.30 | 22.7±40.4 |
| O1 vs O2 | 0.94 | 7.2±38.6 | 0.99 | 1.4±17.8 | 0.68 | 1.9±54.0 | 0.84 | -3.1±39.0 |
|  | **LVEF** | | **RVEF** | | **LV_mass_ED** | | **LV_mass_ES** | |
|  | ICC | bias±2SD (%) | ICC | bias±2SD (%) | ICC | bias±2SD (g) | ICC | bias±2SD (g) |
| CNN vs GT | 0.99 | 1±8 | 0.95 | -2±15 | 0.92 | -11.7±19.3 | 0.92 | -8.8±19.1 |
| Circle vs GT | 0.11 | -7±44 | 0.55 | 1±58 | 0.54 | 27.0±62.3 | 0.21 | 22.2±72.8 |
| O1 vs O2 | 0.99 | 3±9 | 0.93 | 3±21 | 0.79 | 3.4±50.9 | 0.72 | 6.8±52.1 |

*LVEDV: left ventricular end-diastolic volume; LVESV: left ventricular end-systolic volume; RVEDV: right ventricular end-diastolic volume; RVESV: right ventricular end-systolic volume; LVEF: left ventricular ejection fraction; RVEF: right ventricular ejection fraction; LVmassED: myocardial mass in diastole; LVmassES: myocardial mass in systole; ICC: intraclass correlation coefficient.*

**Table 3:** External validation: correlations between CNN and manual gold standard on images with artifacts.

| LVEDV | | LVESV | | RVEDV | | RVESV | |
| --- | --- | --- | --- | --- | --- | --- | --- |
| ICC | bias±2SD (mL) | ICC | bias±2SD (mL) | ICC | bias±2SD (mL) | ICC | bias±2SD (mL) |
| 0.92 | 3.9±23.5 | 0.95 | 3.6±20.8 | 0.83 | 8.0±27.1 | 0.89 | 7.4±22.6 |
| LVEF | | **RVEF** | | **LV_mass_ED** | | **LV_mass_ES** | |
| ICC | bias±2SD (%) | ICC | bias±2SD (%) | ICC | bias±2SD (g) | ICC | bias±2SD (g) |
| 0.98 | -1±11 | 0.93 | -2±17 | 0.91 | -9.2±15.1 | 0.94 | -6.1±15.4 |

*LVEDV: left ventricular end-diastolic volume; LVESV: left ventricular end-systolic volume; RVEDV: right ventricular end-diastolic volume; RVESV: right ventricular end-systolic volume; LVEF: left ventricular ejection fraction; RVEF: right ventricular ejection fraction; LVmassED: myocardial mass in diastole; LVmassES: myocardial mass in systole; ICC: intraclass correlation coefficient.*

Supplementary Images

**Figure 1:** Encoder module

For the encoder module, there are two repeated 3x3 Convolutional layers (zero padding) with stride 1, rectified linear unit (ReLU) activation function and Batch Normalization, a 2x2 Max-pooling layer with stride 2 and a Dropout layer with a dropout rate of 0.3 to regularize the network. As input size is reduced by Max-pooling, after each encoder module, the number of filters is doubled, from 48 (first layer) to 768 (bottom layer) to avoid bottleneck (information loss).


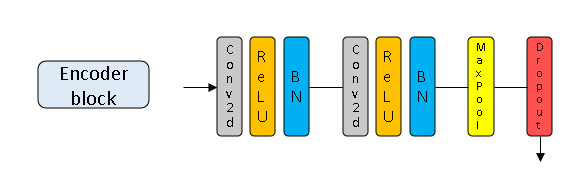


**Figure 2:** Decoder module

The decoder module first up-samples the features map suing an up-sampling operation, reducing the feature channels by half and concatenating with the corresponding feature maps from the encoder. Then a sequence of two 3x3 convolution operation (zero padding) with stride 1, rectified linear unit (ReLU) activation function and Batch Normalization is performed. Similar to the Encoder, this succession of up-sampling and two convolution operations is repeated four times, halving the number of filters in each stage.

**
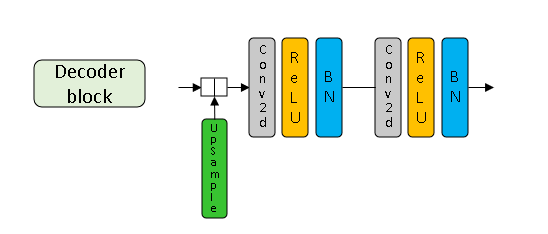
**

**Figure 3:** The attention gate module

Input features $x_{i,c}^{l}$ are weighted with attention coefficients $\alpha_{i,c}^{l}$ computed in attention gate. $x_{i,c}^{l}$is the pixel *i* in layer *l* for class *c*. Attention coefficients, $\alpha_{i}$ ∈ [0,1], identify salient spatial regions in the image and attenuate features responses to preserve only the activations significant to the current task. A gating vector $g_{i}$ is used for each pixel *i* to determine relevant regions. The gating vector contains contextual information to reduce lower-level feature responses. The features-maps of the input tensors $x$ and $g$ are linearly transformed using channel-wise 1x1x1 convolutions and concatenated. Softmax activation function is used to normalize the attention coefficients. All parameters are trained with the standard back-propagation algorithm.
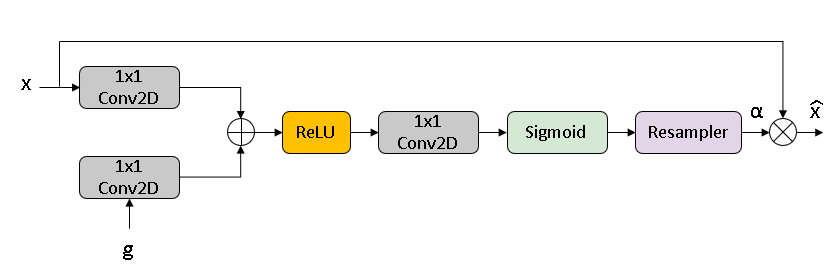


**Figure 4:** Results of correlation and Bland-Altman analysis of automated measurements versus manual measurement on cases without artifacts. Dashed line = bias; solid line = ±2 standard deviations.


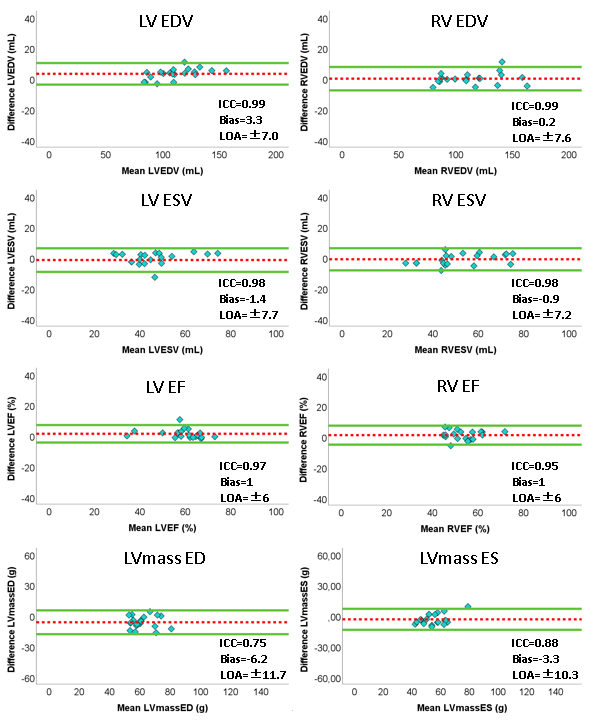

Supplement: Supplementary file 1 — Additional file 1. Table S1. Correlations between CNN and manual gold standard on the artifacts-free images. Table S2. Internal validation: correlations on images with artifacts for the proposed CNN and the commercial software (Circle) in respect to the manual gold standard (GT). Also, the results relevant to interobserver variability between O1 and O2 reported for comparison. Table S3. External validation: correlations between CNN and manual gold standard on images with artifacts. Figure S1. Encoder module. Figure S2. Decoder module. Figure S3. The attention gate module. Figure S4. Results of correlation and Bland-Altman analysis of automated measurements versus manual measurement on cases without artifacts. [file 12968_2022_899_MOESM1_ESM.docx]
